# Simultaneity of health risk factors among Brazilian workers

**DOI:** 10.47626/1679-4435-2026-1578

**Published:** 2026-05-14

**Authors:** Murillo Santos Souza, Ismar Eduardo Martins-Filho, Saulo Vasconcelos Rocha, Raildo da Silva Coqueiro, Carolina Rego Chaves Dias

**Affiliations:** 1 Universidade Estadual do Sudoeste da Bahia (UESB), Programa de Pós-Graduação em Enfermagem e Saúde (PPGES), Jequié, BA, Brazil; 2 Universidade Estadual do Sudoeste da Bahia (UESB), Programa de Pós-Graduação em Educação Física (PPGEF), Jequié, BA, Brazil; 3 Universidade Federal da Bahia (UFBA), Instituto de Saúde Coletiva (PPGSC/ISC), Programa de Pós-Graduação em Saúde Coletiva, Salvador, BA, Brazil

**Keywords:** occupational health, health risk behaviors, sociodemographic factors, epidemiology, chronic noncommunicable diseases.

## Abstract

**Introduction:**

Health indicators are essential tools and, when analyzed jointly, can guide occupational health policies.

**Objectives:**

To analyze the prevalence and demographic variables associated with the simultaneity of health risk factors among Brazilian workers.

**Methods:**

This is a cross-sectional epidemiological study developed based on data from the 2019 National Health Survey. A total of 28,188 formal workers aged 15 years or older were included. Four risk behaviors were analyzed: smoking, alcohol consumption, leisure-time physical inactivity, and inadequate consumption of fruits and vegetables. Multiple binary and multinomial logistic regression models were used, adjusted for sociodemographic variables.

**Results:**

Only 8.2% of workers had no risk factors, whereas 39.7% had two factors and 24.0% had three or four simultaneously. Male workers, those with lower educational levels, and those who were single or divorced had higher odds of simultaneous exposure to risk factors. The probability of exposure progressively decreased in older age groups.

**Conclusions:**

The findings reinforce the importance of intersectoral strategies aimed at health promotion and the prevention of chronic noncommunicable diseases in the workplace, especially among more vulnerable groups.

## INTRODUCTION

Work can be characterized as a fundamental condition of human existence, insofar as individuals relate to nature, construct their reality, and experience social coexistence [^1^]. In this context, workers’ bodies are influenced by the type of work performed, the management model, the division of tasks, and the way work is organized [^2^]. The configuration of these aspects can directly affect workers’ health.

Ongoing economic, political, and social transformations in the world of work have led to significant changes in the relationship between health and work [^3^]. In this scenario, work activity has largely been configured as a source of illness and death, due to the various forms of exploitation to which workers have been subjected [^4^].

In this context, there has been a significant increase in the number of workers on sick leave for health-related reasons [^5^]. In addition, previous studies have reported a high vulnerability among Brazilian workers to exposure to risk factors such as smoking, alcohol consumption, physical inactivity, and excessive intake of high-calorie and ultra-processed foods, which increases the likelihood of developing chronic diseases [^6^].

This scenario highlights the need for actions aimed at the prevention and promotion of workers’ health in Brazil. Among these actions, the screening of modifiable risk factors stands out. Screening these factors is a relevant strategy for assessing, monitoring, and guiding health actions, supporting evidence-based decision-making [^7^]. Furthermore, the simultaneous analysis of these factors may contribute to understanding their clustering patterns and support the development of occupational health policies.

Therefore, the aim of the present study was to analyze the prevalence and the association between sociodemographic variables and the simultaneity of health risk factors among Brazilian workers.

## METHODS

This is a cross-sectional epidemiological study using secondary data from the National Health Survey (PNS for short, in Portuguese) 2019. The project was submitted for review by the National Research Ethics Commission of the Ministry of Health (MH) and was approved in August of the same year under Opinion No. 3,529,376 [^8^].

The PNS is a population-based household survey conducted by the MH in partnership with the Brazilian Institute of Geography and Statistics (IBGE for short, in Portuguese) [^8^]. Data were accessed through the IBGE website and are publicly available. Information was collected through household interviews conducted in different regions of the country between August 2019 and March 2020.

The sampling process was carried out using a three-stage cluster design. The first stage corresponded to the primary sampling units (PSUs). The second stage consisted of the random selection of a fixed number of households within each PSU. The third stage included residents aged 15 years or older, with one individual selected per household. Areas with specific populations, such as Indigenous villages, barracks, military bases, and collective housing units, were excluded. Detailed information on the sampling process and data collection methods can be found in the PNS 2019 report [^9^]. The final sample consisted of 28,188 formal workers.

The PNS 2019 used structured questionnaires to enable comparisons with the 2013 edition and to allow monitoring of health indicators over time. In the present study, information related to household residents was analyzed, including social, economic, demographic, and lifestyle characteristics.

Interviews were conducted in person by trained data collectors, who presented the objectives and procedures of the study to participants. The household respondent and all residents were identified, as well as the adult selected to answer the individual questionnaire, chosen through random selection using a mobile data collection device. Interviews were scheduled at convenient times, with the possibility of two or more visits to the household. Further details are available in a specific PNS 2019 document [^10^].

The variable “simultaneity of health risk factors” was defined as the co-occurrence of two or more risk behaviors in the same individual, considering dichotomous exposure (presence/absence) for each indicator, according to PNS 2019 criteria. The following behaviors were included: leisure-time physical inactivity, inadequate consumption of fruits and/or vegetables, alcohol consumption, and smoking.

To construct the aggregated variable, all factors were assigned equal weight, since the objective was to analyze the coexistence of risk behaviors rather than to develop a weighted severity score. Simultaneity was operationalized through the simple sum of present behaviors (ranging from 0 to 4), which was subsequently categorized according to the number of exposures.

The variables analyzed were: i) sociodemographic characteristics, including sex (male/female), age group (< 30; 30-39; 40-49; ≥ 50 years), education level (incomplete elementary; complete elementary; high school; higher education), race/color (White/Asian; Black; Brown), and marital status (married; divorced; widowed; single); and ii) health risk factors, including smoking (yes/no), alcohol consumption (yes/no), leisure-time physical activity (yes/no), and inadequate consumption of fruits and/or vegetables (yes/no).

Smoking was identified through self-reported current use of tobacco products. A current smoker was defined as an individual who reported using any tobacco product at the time of the interview (e.g., manufactured cigarettes, hand-rolled cigarettes, cigars, pipes, and hookah), regardless of frequency or intensity. For alcohol consumption, the parameter adopted was the intake of five or more drinks on a single occasion for both sexes [^10^].

Individuals were considered physically active during leisure time if they engaged in physical activity outside school or work for at least 150 minutes per week (moderate activities) or 75 minutes per week (vigorous activities). Examples of moderate activities include walking, weight training, water aerobics, and dancing; vigorous activities include running, basketball, soccer, aerobic exercise, and tennis. Leisure-time physical inactivity was defined as not meeting the recommended minimum levels. The PNS 2019 defines adequate consumption as the intake of fruits and vegetables on at least 25 occasions per week [^10^].

To estimate the prevalence of health risk indicators, both individually and simultaneously, frequency distributions and their respective 95% confidence intervals (95%CI) were used. Associations between risk factors (isolated and combined), which are dependent variables, and sociodemographic determinants, which are independent variables, were analyzed using multiple binary logistic regression models, adjusted for all independent variables.

For the “simultaneity” variable, multiple multinomial logistic regression was used, adjusted for all independent variables, with the absence of risk behaviors as the reference category. The significance level adopted was 5% (α = 0.05). Analyses were performed using IBM SPSS Statistics for Windows, version 21.0 (IBM Corp., Armonk, N.Y., USA).

## RESULTS

A total of 28,188 formal workers were evaluated. Most were male (55.5%), aged 30 to 39 years (29.7%), of Brown race/color (48.6%), single (50.1%), and had completed high school education (39.0%). The sociodemographic characteristics stratified by sex are presented in Table 1.

**Table 1:** Sociodemographic distribution stratified by sex among Brazilian formal workers. National Health Survey, Brazil, 2019

Variable	Male		Female		Total	
	n	% (95%CI)	n	% (95%CI)	n	% (95%CI)
Age group, years						
< 30	3,745	23.9 (23.2-24.6)	2,677	21.3 (20.6-22.0)	6,422	22.8 (22.3-23.3)
30-39	4,645	29.7 (29.0-30.4)	3,731	29.7 (28.9-30.1)	8,376	29.7 (29.2-30.2)
40-49	3,682	23.5 (22.8-24.2)	3,195	25.5 (24.7-26.3)	6,877	24.4 (23.9-24.9)
≥ 50	3,573	22.8 (22.1-23.5)	2,940	23.4 (22.7-24.2)	6,513	23.1 (22.6-23.6)
Race/color						
White/Asian	5,775	36.9 (36.1-37.7)	5,125	40.9 (40.0-41.8)	10,900	38.7 (38.1-39.3)
Black	2,088	13.3 (12.8-13.9)	1,496	11.9 (11.3-12.5)	3,584	12.7 (12.3-13.1)
Brown	7,779	49.7 (48.9-50.5)	5,920	47.2 (46.3-48.1)	13,699	48.6 (48.0-49.2)
Marital status						
Married	6,788	43.4 (42.6-44.2)	4,186	33.4 (32.6-34.2)	10,974	38.9 (38.3-39.5)
Divorced	931	6.0 (5.6-6.4)	1,601	12.8 (12.2-13.4)	2,532	9.0 (8.7-9.3)
Widowed	138	0.9 (0.8-1.1)	427	3.4 (3.1-3.7)	565	2.0 (1.8-2.2)
Single	7,788	49.8 (49.0-50.6)	6,329	50.5 (49.6-51.4)	14,117	50.1 (49.5-50.7)
Education						
Incomplete elementary	1,109	7.1 (6.7-7.5)	496	4.0 (3.7-4.4)	1,605	5.7 (5.4-6.0)
Complete elementary	4,050	25.9 (25.2-26.6)	1,826	14.6 (14.0-15.2)	5,876	20.8 (20.3-21.3)
High school	6,239	39.9 (39.1-40.7)	4,756	37.9 (37.1-38.8)	10,995	39.0 (38.4-39.6)
Higher education	4,247	27.1 (27.0-28.4)	5,465	43.6 (42.7-44.5)	9,712	34.5 (33.9-35.1)

95%CI: 95% confidence interval.

Regarding health risk factors, 11.8% (95% CI: 11.4%-12.2%) reported current smoking, 52.2% (95% CI: 51.6%-52.8%) reported alcohol consumption, 51.4% (95% CI: 50.8%-52.0%) were classified as physically inactive during leisure time, and 68.9% (95% CI: 68.4%-69.4%) reported inadequate consumption of fruits and/or vegetables.

Multiple regression analysis indicated that male individuals had higher odds of exposure to smoking, alcohol consumption, and inadequate consumption of fruits and/or vegetables, as well as a lower probability of leisure-time physical inactivity. Workers aged ≥ 50 years and those aged 30-39 years showed a higher likelihood of exposure to smoking and alcohol consumption, respectively, compared with individuals aged < 30 years. Older individuals showed a higher propensity for leisure-time physical inactivity and a lower frequency of inadequate consumption of fruits and/or vegetables (Table 2).

**Table 2:** Multiple logistic regression for health risk behaviors among Brazilian formal workers. National Health Survey, Brazil, 2019

Variable	Smoking			Alcohol consumption			Leisure-time physical inactivity			Inadequate consumption of fruits and/or vegetables		
	%	OR^*^ (IC95%)	p	%	OR^*^ (IC95%)	p	%	OR^*^ (IC95%)	p	%	OR^*^ (IC95%)	p
Sex												
Male	14.6	1.75 (1.61-1.90)	< 0.001	59.5	2.19 (2.08-2.30)	< 0.001	48.9	0.60 (0.57-0.64)	< 0.001	75.2	1.71 (1.62-1.80)	< 0.001
Female	8.3	1.00		43.0	1.00		54.6	1.00		61.0	1.00	
Age group, year												
< 30	11.4	1.00		55.7	1.00		43.2	1.00		78.4	1.00	
30-39	10.5	1.04 (0.94-1.16)	0.462	54.7	1.12 (1.05-1.20)	0.001	48.3	1.23 (1.15-1.32)	< 0.001	71.2	0.72 (0.67-0.78)	< 0.001
40-49	11.4	1.09 (0.97-1.22)	0.139	50.4	1.00 (0.93-1.08)	0.998	54.4	1.42 (1.32-1.53)	< 0.001	66.5	0.56 (0.51-0.61)	< 0.001
≥ 50 Race/color	14.2	1.29 (1.14-1.45)	< 0.001	47.3	0.94 (0.87-1.02)	0.129	60.5	1.61 (1.48-1.74)	< 0.001	59.1	0.38 (0.35-0.41)	< 0.001
White/Asian	11.1	1.00		55.0	1.00		48.6	1.00		63.0	1.00	
Black	13.6	1.01 (0.90-1.14)	0.860	52.6	0.85 (0.79-0.92)	< 0.001	52.6	1.00 (0.92-1.08)	0.907	72.8	1.34 (1.23-1.46)	< 0.001
Brown	11.8	0.89 (0.82-0.97)	0.005	49.8	0.77 (0.73-0.81)	< 0.001	53.4	1.06 (1.00-1.11)	0.051	72.6	1.32 (1.25-1.40)	< 0.001
Marital status												
Married	7.9	1.00		45.0	1.00		51.4	1.00		65.6	1.00	
Divorced	12.8	2.00 (1.74-2.30)	< 0.001	52.4	1.67 (1.53-1.83)	< 0.001	51.8	0.88 (0.80-0.96)	0.006	60.8	1.07 (0.97-1.17)	0.161
Widowed	15.4	2.02 (1.58-2.59)	< 0.001	38.2	1.15 (0.96-1.38)	0.128	62.1	0.86 (0.72-1.04)	0.124	54.3	0.85 (0.71-1.02)	0.074
Single	14.5	2.19 (2.00-2.39)	< 0.001	58.2	1.86 (1.76-1.97)	< 0.001	51.0	1.02 (0.97-1.08)	0.478	73.5	1.21 (1.14-1.29)	< 0.001
Education												
Incomplete elementary	22.3	3.34 (2.86-3.89)	< 0.001	42.7	0.58 (0.52-0.65)	< 0.001	80.9	7.33 (6.69-8.41)	< 0.001	74.2	2.65 (2.34-3.01)	< 0.001
Complete elementary	20.2	3.06 (2.75-3.41)	< 0.001	53.4	0.82 (0.77-0.88)	< 0.001	69.1	4.36 (4.05-4.69)	< 0.001	78.2	2.39 (2.21-2.58)	< 0.001
High school	10.3	1.49 (1.35-1.66)	< 0.001	51.2	0.81 (0.76-0.86)	< 0.001	51.1	2.01 (1.90-2.13)	< 0.001	72.4	1.64 (1.54-1.74)	< 0.001
Higher education	6,6	1,00		54,1	1,00		36,3	1,00		58,3	1,00	

* Adjusted for all independent variables. OR: odds ratio; 95%CI: 95% confidence interval.

Individuals of Black and Brown race/color had a lower probability of alcohol consumption and a higher probability of inadequate consumption of fruits and/or vegetables compared with those of White/Asian race/ color. In addition, workers of Brown race/color had a lower likelihood of smoking (Table 2).

Divorced workers showed a higher prevalence of smoking and alcohol consumption, as well as a lower probability of leisure-time physical inactivity, compared with married individuals. Widowed individuals had higher odds of smoking, whereas single individuals showed a higher likelihood of exposure to smoking, alcohol consumption, and inadequate consumption of fruits and/or vegetables (Table 2).

Individuals with lower educational levels (incomplete elementary, complete elementary, and high school) had higher odds ratios for smoking, leisure-time physical inactivity, and inadequate consumption of fruits and/or vegetables compared with those with higher education, as well as a lower probability of alcohol consumption (Table 2).

When considering the clustering of indicators, 8.2% (95%CI: 7.9%-8.5%) of workers had no risk factors; 28.1% (95%CI: 27.6%-28.6%) had at least one risk behavior; 39.7% (95%CI: 39.1%-40.3%) had two behaviors; and 24.0% (95%CI: 23.5%-24.5%) had three or four behaviors simultaneously.

Among workers who had none of the evaluated risk factors, there was a predominance of females (61.6%), individuals aged 30-49 years (55.7%), those of Brown race/color (45.5%), married individuals (48.0%), and those with higher education (52.4%). In contrast, the lowest frequencies of absence of risk factors were observed among males (38.4%), individuals younger than 30 years (16.4%), widowed individuals (3.0%), individuals of Black race/color (11.0%), and those with incomplete elementary education (2.7%).

Multiple logistic regression analyses of the six possible combinations of two risk factors are presented in Table 3. Male workers showed a higher likelihood of combined exposure to smoking and alcohol consumption; smoking and leisure-time physical inactivity; smoking and inadequate consumption of fruits and/or vegetables; alcohol consumption and leisure-time physical inactivity; and alcohol consumption and inadequate consumption of fruits and/or vegetables. In contrast, for the combination of leisure-time physical inactivity and inadequate consumption of fruits and/or vegetables, a higher probability was observed among females.

**Table 3:** Multiple logistic regression for combinations of two health risk behaviors among Brazilian formal workers. National Health Survey, Brazil, 2019

Variable	Smoking + alcohol consumption			Smoking + leisure-time physical inactivity			Smoking + inadequate consumption of fruits and/or vegetables		
	%	OR^*^ (95%CI)	p	%	OR^*^ (95%CI)	p	%	OR^*^ (95%CI)	p
Sex												
Male	11.4	2.04 (1.86-2.24)	< 0.001	9.4	1.42 (1.30-1.58)	< 0.001	12.3	1.99 (1.82-2.19)	< 0.001
Female Age group, years	5.6	1.00		5.7	1.00		5.8	1.00	
< 30	9.7	1.00		5.6	1.00		9.5	1.00	
30-39	8.5	1.00 (0.89-1.12)	1.000	6.2	1.25 (1.09-1.45)	0.002	8.7	1.05 (0.93-1.18)	0.453
40-49	8.2	0.94 (0.83-1.07)	0.378	8.4	1.57 (1.36-1.82)	< 0.001	9.0	1.02 (0.90-1.16)	0.754
≥ 50	8.9	1.00 (0.87-1.15)	0.999	11.1	1.90 (1.63-2.21)	< 0.001	10.7	1.14 (1.00-1.30)	0.060
Race/color									
White/Asian	8.3	1.00		7.0	1.00		8.3	1.00	
Black	10.4	1.03 (0.90-1.17)	0.666	8.9	0.97 (0.84-1.12)	0.664	11.2	1.09 (0.96-1.23)	0.210
Brown	8.8	0.87 (0.79-0.95)	0.003	8.0	0.91 (0.82-1.00)	0.061	9.9	0.97 (0.88-1.06)	0.481
Marital status									
Married	5.3	1.00		5.4	1.00		6.3	1.00	
Divorced	9.6	2.31 (1.97-2.71)	< 0.001	8.3	1.70 (1.43-2.01)	< 0.001	9.2	1.84 (1.57-2.16)	< 0.001
Widowed	9.0	2.02 (1.48-2.75)	< 0.001	10.8	1.55 (1.16-2.08)	0.003	9.7	1.62 (1.20-2.19)	0.002
Single	11.4	2.37 (2.14-2.63)	< 0.001	9.3	2.11 (1.89-2.34)	< 0.001	11.9	2.17 (1.97-2.40)	< 0.001
Education									
Incomplete elementary	12.7	2.26 (1.88-2.72)	< 0.001	20.4	6.67 (5.55-8.01)	< 0.001	18.8	4.20 (3.54-4.99)	< 0.001
Complete elementary	14.6	2.52 (2.24-2.84)	< 0.001	15.6	5.44 (4.71-6.27)	< 0.001	17.1	3.66 (3.23-4.13)	< 0.001
High school	8.1	1.38 (1.23-1.55)	< 0.001	6.0	2.10 (1.82-2.43)	< 0.001	8.3	1.73 (1.54-1.95)	< 0.001
Higher education Sex	5.5	1.00		2.9	1.00		4.5	1.00	
Male	27.2	1.30 (1.22-1.38)	< 0.001	44.9	2.27 (2.15-2.39)	< 0.001	39.8	0.86 (0.82-0.91)	< 0.001
Female Age group, years	20.5	1.00		26.6	1.00		37.7	1.00	
< 30	21.8	1.00		43.9	1.00		36.4	1.00	
30-39	23.9	1.25 (1.16-1.36)	< 0.001	40.1	1.00 (0.93-1.07)	0.913	38.2	1.10 (1.03-1.19)	0.006
40-49	24.9	1.28 (1.17-1.39)	< 0.001	33.8	0.77 (0.72-0.83)	< 0.001	40.3	1.10 (1.02-1.19)	0.011
≥ 50	26.2	1.33 (1.21-1.45)	< 0.001	28.5	0.62 (0.57-0.67)	< 0.001	40.8	1.00 (0.92-1.08)	0.949
Race/color									
White/Asian	23.5	1.00		35.2	1.00		34.4	1.00	
Black	24.8	0.89 (0.81-0.97)	0.010	39.2	1.03 (0.95-1.12)	0.444	41.3	1.12 (1.03-1.22)	0.006
Brown	24.5	0.90 (0.85-0.96)	0.001	37.3	0.96 (0.91-1.02)	0.162	41.8	1.16 (1.10-1.23)	< 0.001
Marital status									
Married	20.2	1.00		29.7	1.00		37.5	1.00	
Divorced	24.4	1.37 (1.24-1.52)	< 0.001	32.4	1.53 (1.39-1.69)	< 0.001	35.4	0.91 (0.83-1.00)	0.055
Widowed	22.1	1.02 (0.83-1.26)	0.854	22.5	1.10 (0.90-1.36)	0.354	36.6	0.71 (0.59-0.86)	< 0.001
Single	27.3	1.60 (1.50-1.71)	< 0.001	43.6	1.79 (1.69-1.90)	< 0.001	40.7	1.11 (1.05-1.18)	< 0.001
Education									
Incomplete elementary	34.1	2.49 (2.20-2.82)	< 0.001	32.6	1.11 (0.98-1.26)	0.089	62.1	5.40 (4.79-6.07)	< 0.001
Complete elementary	35.6	2.68 (2.48-2.90)	< 0.001	43.2	1.38 (1.29-1.49)	< 0.001	56.1	3.92 (3.64-4.21)	< 0.001
High school	23.8	1.60 (1.49-1.72)	< 0.001	38.1	1.14 (1.08-1.21)	< 0.001	38.7	1.91 (1.80-2.03)	< 0.001
Higher education	16.1	1.00		32.0	1.00		24.8	1.00	

* Adjusted for all independent variables. OR: odds ratio; 95%CI: 95% confidence interval.

A higher combined occurrence of smoking and leisure-time physical inactivity, as well as alcohol consumption and leisure-time physical inactivity, was observed among workers in older age groups. Individuals aged 30-39 years and 40-49 years showed a higher probability of combined exposure to leisure-time physical inactivity and inadequate consumption of fruits and/or vegetables compared with those younger than 30 years. On the other hand, workers aged 40-49 years and ≥ 50 years showed lower combined exposure to alcohol consumption and inadequate consumption of fruits and/or vegetables (Table 3).

Individuals of Black and Brown race/color showed lower odds ratios for the combination of alcohol consumption and leisure-time physical inactivity, but a higher probability of exposure to the combination of leisure-time physical inactivity and inadequate consumption of fruits and/or vegetables. In addition, workers of Brown race/color had a lower likelihood of combined exposure to smoking and inadequate consumption of fruits and/or vegetables compared with those of White/Asian race/color (Table 3).

Divorced, widowed, and single workers showed a higher probability of combined exposure to smoking and alcohol consumption; smoking and leisure-time physical inactivity; and smoking and inadequate consumption of fruits and/or vegetables. Divorced and single individuals also showed a higher likelihood of combined exposure to alcohol consumption and leisure-time physical inactivity, as well as to alcohol consumption and inadequate consumption of fruits and/or vegetables. On the other hand, widowed workers showed a lower probability of combined exposure to leisure-time physical inactivity and inadequate consumption of fruits and/or vegetables, whereas single individuals showed a higher probability compared with married individuals (Table 3).

A higher prevalence of the combinations of smoking and alcohol consumption; smoking and leisure-time physical inactivity; smoking and inadequate consumption of fruits and/or vegetables; alcohol consumption and leisure-time physical inactivity; and leisure-time physical inactivity and inadequate consumption of fruits and/ or vegetables was observed among workers with lower educational levels. In addition, individuals with complete elementary education and high school education showed a higher probability of combined exposure to alcohol consumption and inadequate consumption of fruits and/or vegetables compared with those with higher education (Table 3).

Multiple multinomial logistic regression analysis indicated that male workers had a higher probability of exposure to one risk behavior, as well as to the simultaneity of two and three/four risk behaviors. The probability of exposure to one risk behavior and to two simultaneous behaviors progressively decreased in older age groups. In addition, individuals aged 40-49 years and ≥ 50 years showed a lower probability of simultaneity of three/four risk behaviors compared with those aged < 30 years. Divorced and single workers showed higher odds ratios for exposure to one risk behavior, as well as for the simultaneity of two and three/ four behaviors. Furthermore, the probability of exposure to one, two, or three/four risk behaviors progressively increased among workers with lower educational levels. No association was observed between race/color and the simultaneity of health risk behaviors (Table 4).

**Table 4:** Multiple multinomial logistic regression* for simultaneity of health risk behaviors among Brazilian formal workers. National Health Survey, Brazil, 2019

Variable	Three or four behaviors		Two behaviors		One behavior	
	OR^†^ (95%CI)	p	OR^†^ (95%CI)	p	OR^†^ (95%CI)	p
Sex						
Male	2.68 (2.42-2.98)	< 0.001	1.80 (1.64-1.98)	< 0.001	1.58 (1.43-1.74)	< 0.001
Female	1.00		1.00		1.00	
Age group, years						
< 30	1.00		1.00		1.00	
30-39	0.91 (0.78-1.06)	0.208	0.77 (0.67-0.89	< 0.001	0.76 (0.66-0.88)	< 0.001
40-49	0.78 (0.66-0.91)	0.002	0.69 (0.60-0.80)	< 0.001	0.74 (0.64-0.86)	< 0.001
≥ 50	0.61 (0.52-0.72)	< 0.001	0.52 (0.45-0.61)	< 0.001	0.64 (0.54-0.74)	< 0.001
Race/color						
White/Asian	1.00		1.00		1.00	
Black	1.05 (0.89-1.23)	0.590	1.12 (0.96-1.30)	0.155	1.00 (0.86-1.18)	0.967
Brown	0.99 (0.89-1.10)	0.828	1.01 (0.91-1.11)	0.897	1.00 (0.90-1.10)	0.977
Marital status						
Married	1.00		1.00		1.00	
Divorced	1.91 (1.61-2.28)	< 0.001	1.28 (1.09-1.51)	0.002	1.23 (1.04-1.44)	0.014
Widowed	1.12 (0.81-1.54)	0.509	0.92 (0.69-1.23)	0.567	0.95 (0.71-1.28)	0.728
Single	2.51 (2.25-2.81)	< 0.001	1.49 (1.35-1.64)	< 0.001	1.22 (1.09-1.35)	< 0.001
Education						
Incomplete elementary	8.97 (6.76-11.90)	< 0.001	4.03 (3.05-5.31)	< 0.001	1.78 (1.34-2.38)	< 0.001
Complete elementary	6.88 (5.86-8.07)	< 0.001	3.06 (2.62-3.56)	< 0.001	1.52 (1.30-1.78)	< 0.001
High school	2.30 (2.11-2.65)	< 0.001	1.76 (1.59-1.95)	< 0.001	1.23 (1.11-1.36)	< 0.001
Higher education	1.00		1.00		1.00	

* Reference category: no behavior;

† values adjusted for all independent variables. OR: odds ratio; 95%CI: 95% confidence interval.

## DISCUSSION

This study assessed the prevalence and the association between sociodemographic variables and the simultaneity of health risk factors (smoking, alcohol consumption, leisure-time physical inactivity, and inadequate consumption of fruits and/or vegetables) among Brazilian workers.

Among the factors analyzed individually, inadequate consumption of fruits and/or vegetables was the most prevalent, affecting approximately 70% of individuals. Adequate consumption of these foods exerts an important protective effect on health [^10^], contributing to the prevention of chronic noncommunicable diseases (NCDs), such as cardiovascular diseases, cancer, diabetes mellitus, and metabolic syndrome [^11^,^12^].

Recent studies have investigated fruit and vegetable consumption in Brazil and worldwide [^13^,^14^]. In Brazil, population-based investigations have expanded the evidence on dietary patterns. By analyzing data from the Surveillance of Risk and Protective Factors for Chronic Diseases by Telephone Survey 2015, Veiga et al. [^15^] evaluated the temporal trend in fruit and vegetable consumption among adults in Brazilian state capitals and the Federal District from 2008 to 2023.

The prevalence of regular fruit and vegetable consumption (≥ 5 days/week) remained stable over the period, with an average of 34.1% in the general population and across different sociodemographic groups. Between 2008 and 2014, there was an increase in this consumption (from 33% to 36.5%), except among adults aged 25 to 34 years. However, between 2015 and 2023, a decrease in prevalence was observed (from 37.6% to 31.9%), especially among women, individuals aged 35 to 54 years, and those with more than 12 years of education [^15^].

In addressing changes in the population’s dietary patterns, the Brazilian government has implemented strategies such as the Strategic Action Plan for Tackling Chronic Noncommunicable Diseases in Brazil (2011-2022 and 2021-2030), income transfer policies such as the Bolsa Família program, the expansion of Primary Health Care, and the reformulation of the Dietary Guidelines for the Brazilian Population.

However, during the COVID-19 pandemic, a price inversion was observed, in which ultra-processed foods became more economically accessible than fresh or minimally processed foods [^16^]. This scenario is concerning, as it directly affects lower-income populations, with approximately 28% of the Brazilian population experiencing food insecurity between 2020 and 2022 [^17^]. Reduced access to foods with higher nutritional value favors substitution with ultra-processed products, contributing to the increased incidence of NCDs, especially among more vulnerable groups [^16^].

Currently, studies on health risk behaviors should consider the clustering of these factors, since exposure to one behavior may increase susceptibility to others. The deleterious effects of simultaneity are greater than the sum of individual effects, and interventions targeting multiple behaviors tend to be more effective [^18^]. In the present study, approximately 25% of workers presented three or more risk behaviors simultaneously.

Santos et al. [^19^] demonstrated that the simultaneous presence of factors such as excessive salt consumption, physical inactivity, and alcohol consumption increases the likelihood of hypertension. Similarly, our findings corroborate those of Tassitano et al. [^20^], by showing greater exposure to risk behaviors, such as smoking and alcohol consumption, among men and individuals with lower educational levels, as well as their combinations with other factors. These results reinforce the need for intervention strategies that consider the simultaneity of these behaviors, especially among more vulnerable groups.

The COVID-19 pandemic also contributed to increased exposure to risk behaviors, particularly alcohol consumption and smoking. Schäfer et al. [^21^] identified increases of 20% and 30% in these behaviors, respectively, in a sample of approximately 2,000 individuals in southern Brazil. These behaviors were more frequent among individuals with symptoms of depression and stress, as well as among those with low-quality diets, highlighting the interrelationship between mental health and risk behaviors.

Regarding the simultaneity of three or more risk factors (Table 4), a reduction in the probability of exposure was observed with increasing age. This finding may be related to the greater presence of NCD-related comorbidities among older individuals, such as cardiovascular diseases and diabetes, whose treatment includes lifestyle changes such as reducing or ceasing alcohol consumption and smoking, improving diet, and engaging in regular physical activity [^22^].

With regard to education, individuals with higher educational levels showed a marked reduction in the probability of exposure to multiple risk behaviors. Silva et al. [^23^], analyzing data from the 2013 PNS, observed a similar trend, which suggests that the protective effect of education is related to greater access to information and better understanding of health-related content. Wendt et al. [^24^] highlight that inequalities in access to education persist, which directly influence exposure to risk behaviors, particularly smoking and alcohol consumption, reinforcing the need for public policies aimed at reducing these inequalities.

Although literature points to a higher prevalence of abusive alcohol consumption among individuals with lower educational levels and among those of Black and Brown race/color, the results of this study showed a partially different pattern. This difference may be related to the sample profile, composed exclusively of formal workers, which may reflect greater socioeconomic inclusion and attenuate inequalities observed in the general population.

In addition, the PNS defines abusive alcohol consumption based on the occurrence of at least one episode of high intake on a single occasion in the past 30 days, which may not capture chronic consumption patterns or alcohol dependence, often associated with greater social vulnerability. These findings indicate that the association between sociodemographic determinants and alcohol consumption may assume specific characteristics in the context of formal employment, requiring future investigations that consider aspects such as economic sector, working conditions, and employment relationships.

Moreover, the findings of this study extend beyond the epidemiological dimension and assume practical relevance in the field of Occupational Medicine. The high prevalence and simultaneity of risk factors observed reinforce the need for integrated strategies for worker health promotion, overcoming fragmented approaches focused exclusively on the prevention of traditional occupational risks. Scientific evidence indicates that interventions targeting multiple risk behaviors are more effective than isolated actions [^25^].

Within the Brazilian regulatory framework, these results align with the guidelines of the National Policy on Workers’ Health [^26^], which establishes health promotion as a structuring axis of comprehensive worker care. In addition, updates to Regulatory Standard No. 1 (NR-1 for short, in Portuguese) [^27^] and Regulatory Standard No. 7 [^28^] have expanded the perspective of risk management and the Occupational Health Medical Control Program (PCMSO for short, in Portuguese), enabling the incorporation of preventive actions aimed at behavioral determinants associated with NCDs.

The integration between the PCMSO and the Risk Management Program, as provided for in NR-1 [^27^], allows the systematic screening of factors such as smoking, alcohol consumption, physical inactivity, and inadequate diet, integrating them into educational actions, collective interventions, and longitudinal monitoring of workers’ health. A systematic review indicates that structured workplace health promotion programs contribute to the reduction of modifiable risk factors and to improved health outcomes [^29^].

Additionally, integration with worker health surveillance and primary health care, as outlined in national strategies for tackling NCDs [^30^], strengthens comprehensive and intersectoral care. Thus, the results of this study provide support for the planning of permanent institutional policies for worker health promotion, with a multiprofessional approach, a focus on the most vulnerable groups, and alignment with current regulatory guidelines.

This study has as a strength the analysis of data from the 2019 National Health Survey, providing relevant information for the proposal of strategies to mitigate exposure to risk behaviors among workers in Brazil. However, some limitations should be considered. The small sample size of specific groups, such as workers self-declared as Asian (frequency of 0.7%), made it impossible to perform stratified analyses for this category in multivariate models. Grouping with the White category was necessary to ensure statistical stability and precision of estimates in multinomial regressions. Future studies with specific sampling designs or oversampling of these populations are needed to better understand the particularities of this group.

Regarding occupational characterization, the PNS 2019 database did not allow detailed identification of the main economic sectors or types of formal occupations within the adopted scope. Considering that different occupational activities present distinct contexts and exposures, future studies are recommended to investigate the simultaneity of risk behaviors according to occupational categories, productive sectors, and characteristics of the work process, enabling more targeted interventions.

From a clinical and organizational perspective, it is recommended that occupational health services incorporate the systematic screening of risk behaviors into periodic examinations, integrate health promotion actions into the PCMSO, and develop structured collective intervention programs. For primary health care, the importance of integration with worker health surveillance for the longitudinal monitoring of vulnerable groups is emphasized. Future research should investigate the influence of economic sector, work organization, and working conditions on the simultaneity of risk factors, as well as evaluate the effectiveness of interventions in occupational settings.

Furthermore, it is important to consider that the COVID-19 pandemic intensified exposure to risk behaviors, such as smoking, alcohol consumption, physical inactivity, and inadequate dietary patterns. The effects of these changes in the post-pandemic period still need to be systematically evaluated in order to support preventive and corrective strategies. In light of the scenario of global climate change and the risk of new pandemics or zoonoses, long-term actions are required to prevent behavioral changes that may increase the occurrence of NCDs among workers in Brazil.

## CONCLUSIONS

The results of this study demonstrate a high prevalence of health risk behaviors among Brazilian formal workers, particularly inadequate consumption of fruits and vegetables, leisure-time physical inactivity, smoking, and alcohol consumption. A substantial simultaneity of these factors was also observed, especially among men, individuals with lower educational levels, and those in marital statuses other than married.

The association between multiple risk behaviors and sociodemographic variables indicates an unequal pattern of exposure, reflecting social and educational disparities, as well as differences in access to health information.

The analysis of data from the PNS 2019 highlights the urgency of intersectoral surveillance and intervention strategies aimed at preventing NCDs, considering the synergistic effects of the simultaneity of risk factors. Stratification by age group and educational level allows greater precision in identifying priority groups, contributing to the development of more effective and equitable public policies.

Additionally, the impacts of the COVID-19 pandemic on lifestyle habits should be considered in future analyses, given their influence on health practices and the widening of inequalities. In this context, this study contributes to strengthening epidemiological surveillance and improving health promotion actions in the workplace.

## Data Availability

The data supporting the findings of this study are available within the article.
